# Human health risk assessment for microbial pesticides in the EU: challenges and perspectives

**DOI:** 10.1186/s12940-025-01196-1

**Published:** 2025-07-02

**Authors:** Jimena Barrero-Canosa, Julia Ebeling, Elaine F. Kenny, Philip Marx-Stoelting, Norman Paege, Sabrina Feustel, Daniela Morais Leme

**Affiliations:** 1 German Federal Institute for Risk Assessment (BfR), Berlin, Germany; 2 Department of Genetics, Federal University of Paraná (UFPR), Curitiba, PR Brazil

**Keywords:** Human health, Microbial pesticides, Microorganisms, Risk assessment, Pathogenicity, Infectivity, Antimicrobial resistance genes, NAMs

## Abstract

The risk assessment of microbial pesticides in the European Union (EU) is covered by a regulatory framework based on EU Regulation 1107/2009 and 546/2011 together with the data requirements in EU Regulation 283/2013 and 284/2013, Part B, respectively (all amended in 2022). Furthermore, several guidance documents specify the data requirements for the human health assessment. As in other regulatory contexts, the assessment of hazardous properties of a microbial plant protection product (PPP) can be based on in vivo data. In order to decrease the use of test animals, support high-throughput data generation with larger repetition, and to facilitate faster testing methods, New Approach Methodologies (NAMs) for this field need to be developed. Here we focus on the assessment of the potential pathogenicity/infectivity and the presence of transferable antimicrobial resistance (AMR) genes of a microorganism when utilised as the active substance (AS) in a PPP. For the purpose of risk assessment of microbial PPPs, NAMs developed in view of the Next Generation Risk Assessment (NGRA) for chemicals can be applied. However, major drawbacks in the ability to use existing NAMs in the risk assessment of microbial pesticides are the reliability of Adverse Outcome Pathway (AOP) generated data for humans and the practicability of in vitro methods to test living microorganisms. It must be emphasised that tests for risk assessment are only useful if the test interpretation is clearly defined. Without prior definition of the possible effects and their interpretation, including the possible outcome for risk assessment, the test has limited value, as the results may raise more questions than answers. Overall, the regulatory assessment of the human health effects of microbial pesticides used in PPP needs reliable and robust data. These data should ideally be presented by an applicant based on animal-free study setups together with thorough literature searches.

## Introduction

The European Green Deal is a central component of the European Union (EU)’s climate policy. It presents itself as a pan-European effort to make food and production systems more sustainable and robust with minimal side effects on human health, non-target organisms, and the environment [1]. The Farm2Fork Strategy is an important element of the European Green Deal, which aims to reduce the use of chemical and more hazardous pesticides by 50% by the year 2030. It supports the use of biological and physical methods over conventional chemical methods, advocates for measures against the spread of antimicrobial resistance (AMR) and focuses on the development of organic farming and integrated pest management [2]. The use of biopesticides that have been assessed according to the detailed EU regulatory framework is an essential component of the ambitious aims of the European Farm2Fork Strategy.

The term “biopesticide” is not uniformly defined by organisations and legislators. In its broadest interpretation, it means all biologically derived substances: microorganisms (bacteria, algae, protozoa, viruses and fungi), semiochemicals, macroorganisms/invertebrates such as insects and nematodes, and plant extracts/botanicals [3]. In addition, new methods like RNA interference (RNAi) and the use of microbial consortia are under development [4, 5].

In the EU, the evaluation of biopesticides containing microorganisms (i.e., microbial pesticides) is based on specific data requirements aimed at the approval of microbial active substances (AS) at the EU level and the approval of the corresponding products at the Member State level [6]. For microbial AS, data requirements follow a stepwise approach aimed at assessing pathogenicity and infectivity, the presence of antimicrobial resistance genes (AMR) and the toxicity of metabolites. In the case of microbial plant protection products (PPPs), a larger data package is required. This includes studies on acute oral, dermal, and inhalation toxicity, as well as skin and eye irritation, and skin sensitisation. These requirements are also organized in a tiered approach, where animal testing may be waived based on the first two tiers, relying on existing medical data and a weight of evidence (WoE) approach using reliable sources to assess the potential toxicity of the formulation. Only after a microorganism is approved at the EU level can applications for products containing that microorganism be submitted for authorization at the Member State level [6]. Other naturally derived substances such as semiochemicals, plant extracts, microbial metabolites, or RNA are assessed according to the chemical AS framework and, if no tailored requirements are specified for the AS type, by adapting the assessment strategy [7]. In general, in view of the EU Chemicals Strategy for Sustainability, the approval of natural and low-risk AS should be facilitated to reduce the use of conventional chemical pesticides [8].

The approval process will, in the future, make use of risk assessment methodologies according to the principle of the 3Rs (Replacement, Reduction, and Refinement), thus reducing the use of laboratory animals as much as possible. By using methods that are geared to the data actually required for the evaluation and characterised by high reproducibility and informative value, this method will lead to a sound conclusion on the safety of human health and the environment [9].

In the assessment of human health risks associated with microbial AS, the first step can be based on a literature search for the microorganism, and related strains, and the metabolites potentially produced. The WoE approach can be used to demonstrate the potential pathogenicity and infectivity of the microorganism. In vivo animal studies are only be required if the applicant cannot demonstrate the absence of these properties [6]. However, even when performed, the informative value of acute animal studies for critical issues is often limited, as the host specificity of the microorganism might not reveal a pathogenic potential in animals. Therefore, the effects in rats or mice cannot be used to infer effects in humans or other animals with certainty. Examples include the plague, caused by the bacterium *Yersinia pestis*, which can be asymptomatic in rodents but cause severe infections in humans and other animals [10], and the Foot and Mouth Disease Virus (FMDV) which results in highly infective and pathogenic conditions in cattle or sheep but not in humans [11].

In addition, such animal testing would, in most cases, not reveal the acute toxicity of secondary metabolites, as the metabolite of concern would likely need to be present at an effective concentration in the test system to elicit a measurable toxic response. Secondary metabolites of microorganisms are produced under specific physical and biological conditions, as they are not directly involved in primary metabolism but rather play roles in microbial survival and ecological interactions [6].

Unlike conventional chemical AS, where toxicological effects can be directly associated with defined doses under controlled exposure conditions, microbial AS pose additional challenges. The production of secondary metabolites in microbial preparations is highly variable and environment-dependent (e.g., nutrient starvation, host contact, or endophytism). Consequently, the absence of observed effects in animal studies does not necessarily indicate an absence of hazard, but may instead reflect insufficient metabolite production to reach the threshold concentration required to induce acute toxicity. If acute testing does not result in adverse effects, the effects based on repeated, sub-chronic, or chronic exposure would not be investigated, according to the tiered approach usually used in risk assessment. This leads to a lack of information on secondary metabolites, which are not included in the test material because they will be formed only under certain environmental conditions [12, 13].

Moreover, the test guidelines for biopesticides are almost 30 years old, and newer guidelines for chemicals are missing adaptions for microbial AS [14]. Therefore, new strategies for testing and assessing biopesticides are necessary. One approach lies in the Integrated Approaches to Testing and Assessment (IATA) concept based on Adverse Outcome Pathways (AOP) [15]. However, the AOP concept has been mostly designed for chemical toxicology and would need adaption to cover also pathogenicity or infectivity of microbial pesticides, as generally, the microorganism itself is the initiator of the pathogenicity (i.e., the disease it causes) rather than a chemical-biological interaction of a molecular initiating event. While the development of AOPs for the assessment of microbial infections and their associated effects is not as advanced as for chemical stressors, progress is evident through several examples, particularly those established within the framework of the CIAO consortium in response to Coronavirus Disease 19 (COVID-19) – an initiative of the Joint Research Centre of the European Commission [16]. Among the notable outcomes of this initiative was the development of AOPs through the systematic collection and critical evaluation of the scientific literature on COVID-19 pathology. In particular, AOP 430 provides mechanistic insight into the interaction between SARS-CoV-2 and host cells required for viral entry and replication, delineating early key events that contribute to the progression of disease outcomes represented within the COVID-19 AOP network [17].

Creating tiered approaches in decision trees could provide a solution to overcome the current difficulties in biopesticide risk assessment. An example of a stepwise approach for natural substances when used in plant protection is shown in Busschers et al. [18]. However, a critical gap remains in defining how information should be generated, selected, and evaluated at each stage of the assessment. Thus, the challenge lies not only in designing a tiered approach aligned with the regulatory data requirements but also in establishing clear and robust criteria for the inclusion and quality assessment of data sources. In summary, the problem in the risk assessment of microbial pesticides is to identify the suitable testing method and to define the criteria for evaluating the data, even if one has to deal with ambiguous results. Similar problems are faced in the risk assessment of all biologically derived active ingredients in the food and feed chain, e.g., plant extracts.

This review article aims to summarise key information regarding the human health risk assessment of microbial pesticides in the EU, with a particular focus on challenges in the authorisation process. It explores potential advancements, including the application of NAMs, to enhance regulatory frameworks and improve assessment efficiency. The primary focus is on data requirements related to pathogenicity, infectivity and AMR genes. The toxicity of secondary metabolites and sensitisation are not covered by this article as they have been extensively reviewed by Paege et al. [19] and Leme et al. (manuscript in preparation) [20], respectively.

## Data requirements for microbial pesticides and the potential use of NAMs to overcome current challenges

**Fig. 1:**
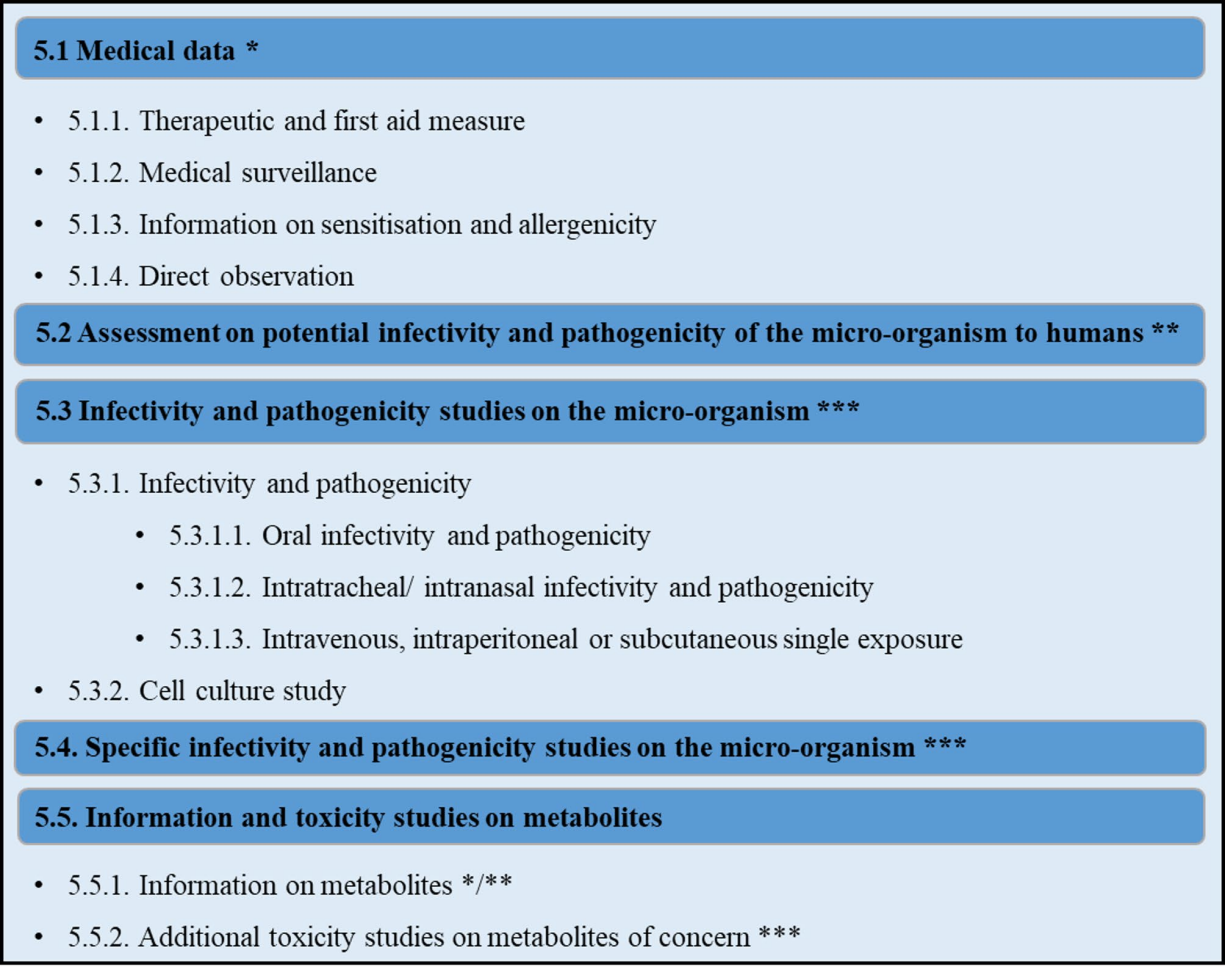
Data requirements concerning effects on human health from microbial active substances (AS) (see data point 5, Reg. (EU) 2022/1439) * Available literature and experimental data are used to address this data point ** WoE approach. Available literature data, expert knowledge or experimental data provided under other points (e.g., 5.1 and 5.5.1) and other reliable sources to justify a possible waiver of further experimental data (see 5.3, 5.4, 5.5.2) to reduce animal testing *** Waiver based on available literature data, expert knowledge or experimental data possible. Additional experimental data according to accepted test guidelines might be required, based on a case-by-case decision

**Fig. 2:**
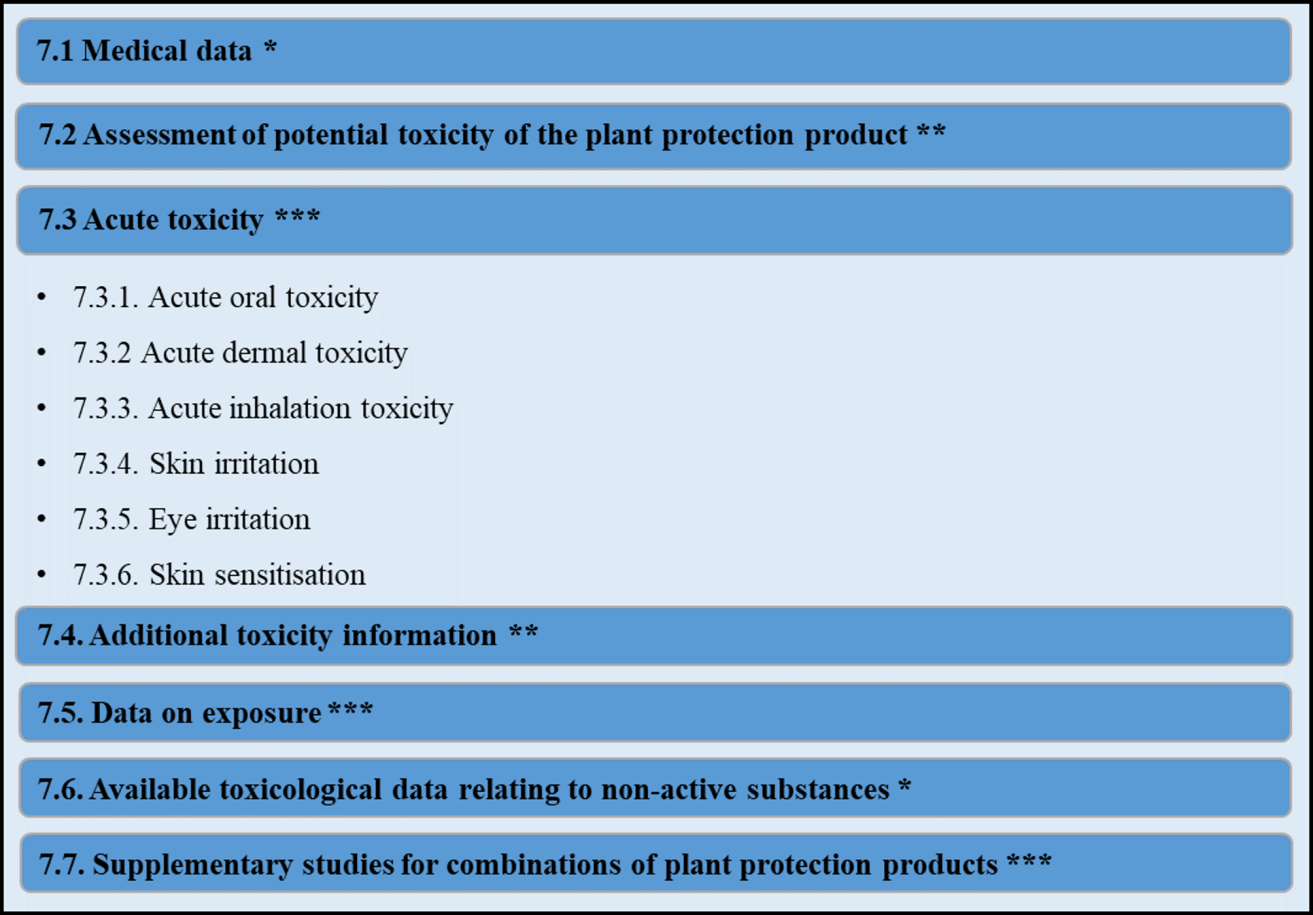
Data requirements concerning effects on human health from microbial plant protection products (PPPs) (see section 7, Reg. (EU) 2022/1440) * Available literature and existing experimental data are used to address this data point ** WoE approach. Available literature and experimental data, expert knowledge, information provided under Sections 2, 3, 4, point 7.1 or retrieved from any other reliable sources (e.g., IATA, Classification, Labelling and Packaging (CLP) calculation rules in accordance with Regulation (EC) No 1272/2008 or read-across data from similar preparations) to justify a possible waiver of further experimental data to reduce animal testing *** Waiver based on available literature data, expert knowledge or experimental data possible. Additional experimental data according to accepted test guidelines might be required, based on a case-by-case decision

At the European level, the EU Commission has created a legal framework for the evaluation of microbial pesticides. While EU Regulation (EC) No 1107/2009 governs the “placing of plant protection products (PPP) on the market”, there are also other regulations on the data requirements for the evaluation of microbial active substances (AS) and the subsequent products [21]. (i) Regulation (EU) 2022/1439 on AS data requirements (Fig. 1) [22], (ii) Regulation (EU) 2022/1440 on product specific data requirements (Fig. 2) [23], (iii) Regulation (EU) 2022/1441 regarding specific uniform principles for evaluation and authorisation of PPP [24] and (iv) Regulation (EU) 547/2011 regarding the labelling requirements provide the legal framework for the evaluation of PPPs containing microbial AS [25]. Specifically, the endpoints relevant for microbial risk assessment of human health are covered by the AS data requirements, mainly presented in the chapters “Biological properties” and “Effects on Human Health” of the Regulation (EU) 2022/1439.

This legal framework is supplemented by the EU via the publication of guidance documents, which assist in the assessment of microbial secondary metabolites or the assessment of antimicrobial resistance (AMR). The guidance documents facilitate greater harmonization of the assessment across the EU member states and also allows more harmonization in the preparation of data by the applicant. For the risk assessment of microbial AS, it is also advisable to look at comparable regulatory areas. In addition to the safety assessment of microorganisms used in PPPs, the European Food Safety Authority (EFSA) also evaluates microorganisms in connection with feed additives, food additives, or food enzymes, and as such important guidance documents can be applicable across disciplines (e.g., in the EFSA Panel on Additives and Products or Substances used in Animal Feed – FEEDAP) [26]. However, as the risk assessment for the use of microbials in food and feed mainly covers the hazards via the oral exposure route, the data requirements for the evaluation of microbial AS understandably focus on a different endpoints.

The hazards related to a microbial AS also vary from those relevant for chemical AS. As microbial AS are living organisms with strain specific properties, pathogenicity and infectivity to humans and animals are important endpoints for the health risk assessment. This must be assessed separately from the microorganism’s capacity to produce metabolites of concern, transfer AMRs or the potential to induce sensitising reactions.

Therefore, the assessment of a microorganism achieves a different level of complexity than with chemical agents. While the composition of the technical grade active ingredient (TGAI) can be determined very precisely using analytical methods as part of an assessment of the manufactured AS produced, the in-situ production of toxicologically relevant secondary metabolites is sometimes a black box.

As with conventional chemical AS, avoiding animal testing is a topic of discussion for the evaluation of microbial AS, combined with the desire to establish more evaluation-relevant data at low cost, high throughput and with a higher information gain. The evaluation of microbial AS relies more on assessing all collected information in a weight of evidence (WoE approach and with a strong focus on existing literature. The guidance document from EFSA, 2011 [27] outlines guidelines for submitting scientific literature when applying for pesticide approval under EU Regulation (EC) No 1107/2009. Thorough relevance and reliability assessment of the literature search results ensure that only scientifically rigorous and applicable studies are used to inform the regulatory approval process.

The relevance criteria for selecting studies focuses on hazard identification and characterization, and exposure assessment for the AS. Developing these criteria is a continual process that involves refining the relevance through initial literature searches, ensuring the inclusion of studies that are most applicable. Importantly, studies not conducted in adherence to Good Laboratory Practice (GLP) may still be considered if they meet the relevance requirements.

The reliability of the studies is assessed based on factors such as peer review, sound study design, appropriate sample size and transparent methodologies. Additionally, studies must be published in peer-reviewed journals indexed in major databases (e.g., Pubmed, Scopus or Web of Science) and free from significant conflicts of interest. Depending on the quality, taking the EFSA guidance document into account, the assessor concludes on the impact of each report to the overall assessment.

Other current tools utilised in the WoE approach for evaluating the safety of microbial pesticides include whole genome sequencing (WGS), biological chemistry identification, pathogenicity, infectivity and toxicity data, and hazard assessments of formulations guided by regulations such as the European Chemicals Agency (ECHA) Classification, Labelling and Packaging (CLP) [28]. While these tools offer a solid foundation, further refinement and development of methodologies specifically tailored to microbial pesticides are essential to address their unique characteristics effectively [6, 19, 28]. That approach is aimed at so-called New Approach Methodologies (NAMs).

The concept of NAMs relies on tiered combinations of in silico tools, in vitro systems, organ models, and omics approaches, integrated with physiologically based toxicokinetic modelling and complex exposure models. NAMs strongly emphasise mechanism-driven approaches rather than traditional apical toxicological endpoints, and by utilising the adverse outcome pathway (AOP framework, NAMs enable the development of unified models for toxicity endpoints, capturing a spectrum of interconnected biological effects [29].

The large increase in the number of NAMs is driven by their potential to improve hazard prediction for risk assessment purposes. In this context, NAMs are central to the transition toward Next Generation Risk Assessment (NGRA, which predominantly relies on NAMs data but can also incorporate in vivo data or histopathology when appropriate. Key benefits of NAMs for regulatory toxicology include their focus on mechanisms of action and improved translation of data to human relevance [9, 29].

Although promising, the adoption of NAMs in regulatory toxicology still faces significant challenges and requires further progress. While several test methods have been developed and adopted by the Organisation for Economic Co-operation and Development (OECD) following successful validation processes, these efforts have largely focused on single chemical substances [30]. Attempts to adapt existing NAM guideline studies for microbial pesticide testing have been made, but substantial gaps remain [28].

Many toxicological endpoints for microorganisms (e.g., taxonomic identification, potential virulence factors) are already addressed today by comparing WGS data with curated databases. Additionally, it enables the identification of genes that confer resistance to antimicrobial compounds of clinical relevance for humans and animals, genes involved in the production of known potentially harmful metabolites like toxins or antimicrobial compounds, and genes that code for virulence factors, lytic or lysogenic properties, or genetic modifications. At the same time, WGS data can be interrogated to exclude the presence of genes that may raise concerns. If gene products of potential concern can be ruled out, no exposure assessment for this substance is required. Here too, the knowledge gained is limited by the data currently available in relevant databases and literature. Improving these databases by integrating all available information, where appropriate using new tools such as machine learning and artificial intelligence, is therefore an important task to facilitate risk assessment of microbial pesticides.

What is ultimately important is the suitability of such NAMs for inclusion in the regulatory assessment and proof that, in addition to positive ethical, financial, quantitative and time-related aspects, a comparable or even greater gain in knowledge can be expected. While complex endpoints, such as the pathogenicity and infectivity of microorganisms, are currently difficult to address using NAMs and without animal experiments or clinical literature data, the assessment of microbial secondary metabolites toxicity is already feasible today [19].

## Pathogenicity and infectivity

The potential for human pathogenicity and infectivity are key elements in the regulatory approval procedure of microbial pesticides in the EU. According to EU regulatory definitions *pathogenicity* denotes the non-opportunistic ability of a microorganism to cause harm and disease to the host, independent of host immune status whereas *infectivity* refers to the ability of a microorganism to enter, survive, and multiply within a host. As such, in the domain of human health, the EU employs a binary, hazard-based approach. Microbial active substances (AS) that are pathogenic and/or infectious to humans are not eligible for approval. This contrasts with risk-based approaches used for non-target environmental organisms, where quantitative risk assessments and exposure limits are applied [22]. The EU evaluation process set out in Commission Regulation (EU) No. 2022/1439 (specifically Part B, Section 5) outlines a stepwise framework for assessing pathogenicity and infectivity for microbial AS. See Table 1 for a brief overview of the information to be assessed in this section.

As such, the risk assessment for pathogenicity and infectivity must address a set of defined data requirements. First and foremost is the precise taxonomic identification of the microorganism to the strain level, which is typically achieved using whole genome sequencing (WGS) (details of which can be found in the antimicrobial resistance (AMR) section). WGS can also detect the presence or absence of genes associated with virulence, toxin production, or AMR (further details below). Another key role of WGS is the ability to carry out comparative genomic analysis against known pathogenic strains, offering insight into genetic similarity and risk potential [31]. The WoE approach is also applied, utilising the biological properties of the microorganism, including the WGS-data analysis, medical data from studies and extensive literature searches to determine the risk to humans. The WoE approach ensures the comprehensive collection and critical evaluation of all available scientific information relevant to the microbial strain, and related strains, under assessment. Based on the outcome of these initial investigations, the necessity for a tiered approach involving in vivo studies is determined.

The current tiered approach relies on classical testing methods using animal models as biological surrogates. Tier 1 evaluation includes in vivo studies in rodents in which the microorganism is administered via the oral, intranasal and intravenous routes at a single high dose. A defined post-exposure observation period, determined by the biological properties of the microorganism, follows. During the course of the observation period blood and faeces are examined. Blood samples are analysed to detect the presence of viable microorganisms or their genetic material, providing evidence of systemic infectivity. Faecal samples are collected to assess the persistence, colonisation potential, and shedding of the microorganism in the gastrointestinal tract. Finally, the animals are sacrificed to assess infectivity and clearance across key target organs such as the lungs, liver, spleen and brain. The selection of key organs is dependent on the microorganism being studied.

If the Tier 1 tests do not conclusively demonstrate the absence of risk to human health, Tier 2 studies must be employed. Tier 2 approaches involve the design and execution of tailored in vivo studies that address the specific uncertainties identified in the Tier 1 studies. They aim to provide further mechanistic insights and reduce residual uncertainty in the risk assessment process [6].

However, both Tiers face limitations, including the lack of validated OECD test guidelines specific to microbial AS, with only “proposed test guidelines” outlined in the OPPTS 885 series [32–37] and the limited availability of non-animal testing models that meet regulatory standards. As such, the inclusion of in vivo animal studies often results in “data gaps” in the risk assessment of pathogenicity and infectivity in the EFSA “Conclusion on Pesticide Peer Review” reports for microbial AS. Examples of data gaps identified include the potential adverse effects after repeated exposure by inhalation to *Bacillus thuringiensi*s subsp. *kurstaki* strain SA-11 [38] and the inconclusive outcome of pathogenicity and infectivity testing of *Aspergillus flavus* MUCL54911 due to study limitations [39].

To address the limitations and ethical concerns associated with in vivo testing, and in accordance with European Directive 2010/63/EU, which legally established the 3Rs principle (Replacement, Reduction, and Refinement) [40], there is increasing focus on the development and regulatory adoption of NAMs. These include advanced in vitro systems, computational models, and other non-animal methods that offer promising pathways to refine or replace animal studies, particularly in the early stages of microbial risk assessment.

In particular, in vitro assays using human-derived cell lines are increasingly employed to assess microbial cytotoxicity, inflammatory responses, and barrier integrity. The EFSA Guidance on *Bacillus* safety, 2014 [41] recommends the use of epithelial cell lines for the detection of cytotoxicity. Moreover, a recent study by Fichant et al.. evaluated 48 *Bacillus* spp. for their potential infectivity/potency in vitro and in vivo [42]. The human intestinal Caco-2 cell line was used to measure cytotoxicity and IL-8 production upon treatment with bacterial culture supernatants. In addition, an in vivo toxicity assay was carried out using *Drosophila melanogaster*. The flies were exposed to *Bacillus* spp. spores via food, and survival rate and intestinal leakage were examined. The in vivo study examined the toxicity of *Bacillus* spp. after traversing the gut and, hence, is representative of a “real-world” scenario unlike the in vitro Caco-2 studies. Overall, the study revealed differences in the toxicity of the species tested depending on the route leading to exposure of the intestinal cells to the strains. The observed differences demonstrate that experiments in an in vivo setting remain central to the risk assessment process and highlight the potential of *D. melanogaster* as a model organism.

In addition, organoid models (three-dimensional structures derived from stem or progenitor cells) have emerged as powerful tools for simulating complex host tissues, including the gut, lungs, and brain. They overcome the limitations of conventional 2D cell culture models, which often fail to replicate the complex cellular interactions and microenvironmental cues essential for organ function. This is particularly relevant for studying microbial infections, as pathogens disrupt finely regulated cellular homeostasis, leading to disease [43, 44]. To further enhance organ-like functionality in vitro, organoid technologies have been integrated with organ-on-a-chip systems, which provide microphysiological environments crucial for understanding complex biological processes such as host-pathogen interactions [43, 45]. For example, the application of mechanical stimulation to lung models to mimic breathing-like cyclic motion has been shown to significantly reduce Influenza A virus infection efficiency [45]. Thus, both organoid and organ-on-a-chip technologies hold great promise for advancing the study of infection dynamics and pathogenesis [43, 45].

Despite their advantages, these models also present certain limitations. While they offer a rapid and cost-effective alternative to traditional 2D cultures and animal models, challenges remain in improving their scalability, reproducibility, and ability to fully recapitulate the complexity of host-pathogen interactions [45]. However, ongoing research efforts are focused on optimising these systems to enhance their predictive power and translational relevance.

Continued refinement of NAMs for assessing pathogenicity and infectivity is essential to improve the efficiency and scientific robustness of microbial pesticide evaluations. These advances also pave the way for addressing other critical safety aspects, such as antimicrobial resistance.

**Table 1:** Key information required to assess pathogenicity and infectivity of microbial active substance (AS)

Data	Purpose
Literature review	Identify reported associations with adverse health outcomes
Taxonomic identification (e.g., WGS)	Establish strain identity; screen for virulence or AMR genes
Comparative genomics	Assess similarity to known pathogenic strains
In vivo studies (Tier 1 and Tier 2)	Determine pathogenicity/ infectivity in animal models

## Antimicrobial resistance (AMR)

**Fig. 3:**
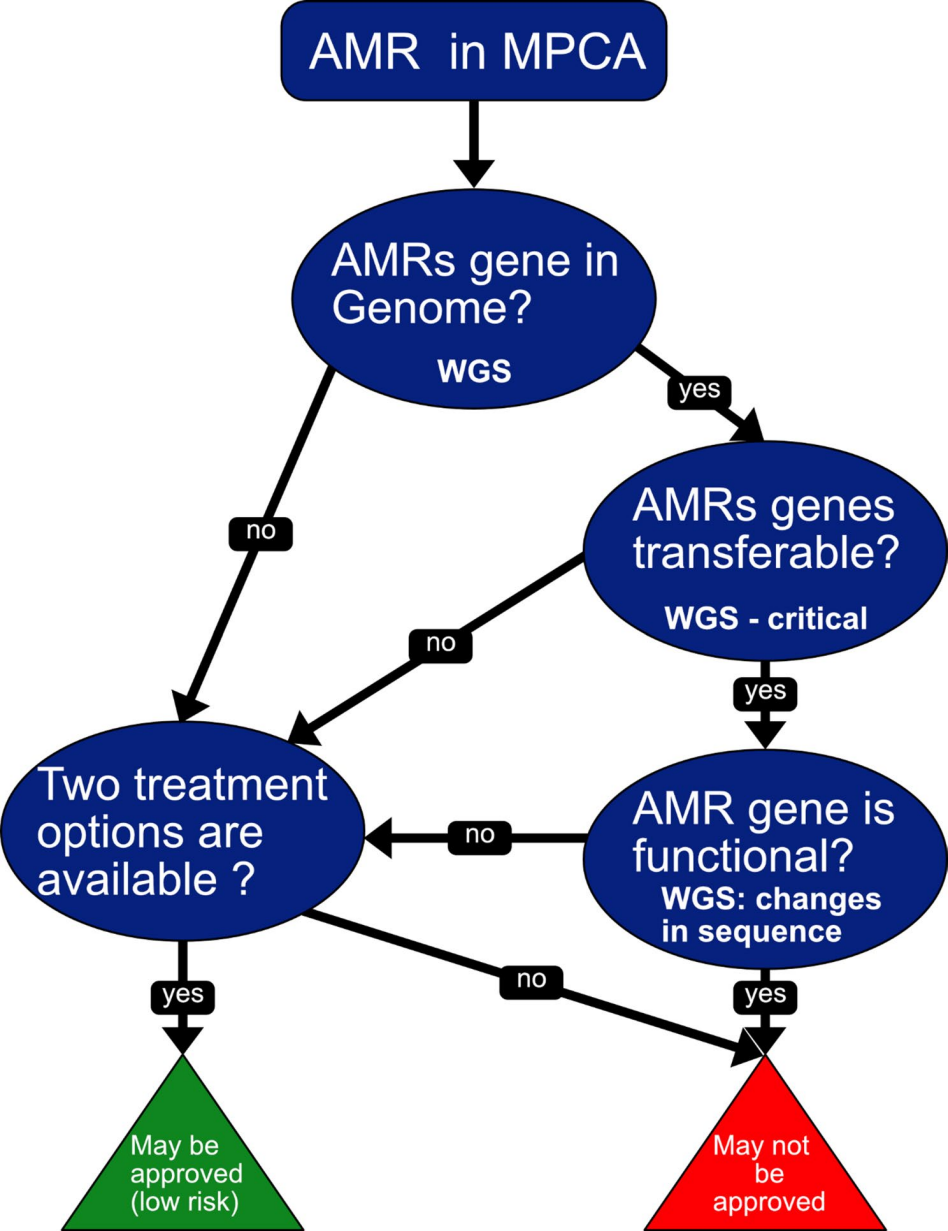
Decision tree related to antimicrobial resistance (AMR) for the approval of a microbial as low-risk AS according to EU Regulation EC No 1107/2009 Annex II (amended by Regulation (EU) 2022/1438)

Assessing microbial pesticides for antimicrobial resistance (AMR), especially those using bacterial strains as microbial pest control agents (MPCAs), is crucial to ensure these strains are not multidrug-resistant and cannot transfer AMR genes to other microorganisms [46]. This concern is heightened by the increasing incidence of infections caused by AMR strains, a significant public health issue emphasized by the World Health Organization [47]. For instance, in 2015, over 600,000 patients in the EU developed infections with multidrug-resistant (MDR) pathogens, leading to at least 33,000 deaths [48]. The risk associated with AMR in microbial pesticides is the potential transfer of resistance mechanisms to native microorganisms of the treated crops, soil, etc. This concern is addressed in the EU through Regulation (EU) 2022/1439 [22], amending Regulation (EC)283/2013 [49], and Regulation (EU) 2022/1441 [24], amending Regulation (EC) 546/2011 [50], stating *that “if resistance to antimicrobials can be transferred to other microorganisms*,* including human and animal pathogens*,* the microorganism should not be approved”*. Furthermore, to be considered a low-risk AS according to Annex II Regulation EC 1107/2009, an active substance (AS) that is a microorganism, other than a virus, must be sensitive to at least two classes of antimicrobials. Thus ensuring that there are at least two possible treatment options in the event of an opportunistic infection. A brief overview of the decision tree is shown in Fig. 3.

In general, most microorganisms like bacteria and fungi produce antimicrobials to defend their ecological niche and accordingly have AMR genes for defense [51–53]. Thus, possessing AMR genes is not a criterion for exclusion of the approval of the microorganism as MPCAs per se. However, the transferability of known functional AMR genes coding for resistance to clinically relevant antimicrobial agents is one cut-off criterion for the approval of microorganisms as AS along with a lack of pathogenicity or infectivity to humans. That means no approval of the strain of a microorganism as MPCA can be granted if it carries a resistance gene which can be transferred to other microorganisms, including human and animal pathogens [21, 54, 55].

In bacteria, the transfer of genetic material can be achieved via multiple chromosomal and extrachromosomal mobile genetic elements (MGE) such as plasmids, bacteriophages, integrative conjugative elements, transposable elements, integrons, gene cassettes or genomic islands [56]. In contrast, in fungi and viruses (excluding bacteriophages from the latter), horizontal gene transfer is considered irrelevant for risk assessment [46]. In fungi, horizontal gene transfer is a very rare event and not associated with specific mechanisms like the listed above for bacteria [57]. However, information on the antimicrobial susceptibility of fungi is still needed, and at least two treatment options for possible opportunistic fungal infections have to be provided for the approval as AS. No concern of horizontal gene transfer is derived from viruses, except bacteriophages. During the lysogenic life cycle of temperate bacteriophages, i.e., the temporary integration of the phage genome into the bacterial host genome, the transfer of AMR genes into the bacterial host genome is possible [58].

The European Commission and Member States have jointly developed SANTE/2020/12,260 guidance to evaluate AMR in MPCAs [46]. The guidance outlines procedures for assessing the risk of exacerbating the spread of AMR, both in human and veterinary contexts, regarding the approval and low-risk criteria established under EU Regulation (EC) No 1107/2009. Additionally, SANTE/2020/12,260 emphasises the utilisation of NAMs, specifically whole genome sequencing (WGS), to assess the potential risk associated with the presence and transfer of AMR genes. It proposes a systematic approach involving WGS to detect genes that could confer microbial resistance. Subsequently, it recommends using bioinformatics tools to predict the likelihood of these genes being transferred to other bacterial strains in the environment. Furthermore, the guidance suggests using traditional culture microbiology methods to assess the bacterial phenotype, specifically identifying which antibiotics the resistant bacteria can withstand. Phenotypic testing with methods determining the minimum inhibitory concentration (MIC) of a selected group of antimicrobials is necessary because the mere presence of the AMR gene does not necessarily mean that the gene is functional.

The decision criteria for the approval of a bacterial strain as MPCA in regard to AMR are briefly summarised here (see also Table 2):

(i) A bacterial strain with an AMR gene identified by WGS but not located on a MGE and without resistance in the phenotypic testing can be approved as a MPCA.
(ii) If an AMR gene has been identified via WGS and also the localization on a MGE has been demonstrated, the strain can only be approved if non-functionality is demonstrated in the phenotypic testing.
(iii) Also, if an AMR gene is identified and resistance has been demonstrated in the phenotypic assay but no association with an MGE has been found, the strain may be approved because the gene is considered intrinsic.
(iv) If no AMR gene was identified via WGS but the phenotypic screening demonstrates resistance, the strain may be approved until further information about this unknown AMR becomes available. However, SANTE/2020/12,260 is a collaborative guidance among Member States and does not carry legally binding implications [46].

In parallel, an EFSA statement on the qualified presumption of safety (QPS) on acquired AMR was published in 2023 [59]. Similar to SANTE/2020/12,260, the EFSA document also suggests a tiered approach, including WGS analysis for the detection of AMR genes to achieve the prevention of the spread of AMR from bacteria used in the food and feed chains, which includes bacteria used as MPCAs in PPP. In terms of terminology, the EFSA statement distinguishes between acquired and intrinsic AMR genes, which accordingly goes in line with the terms transferable and non-transferable functional AMR genes in the SANTE/2020/12,260 guidance document.

WGS is increasingly used to detect AMR genes and their transferability in fields like pest control and in monitoring and preventing infections [60]. Two main factors contributing to WGS’s appeal are the advancements in sequencing technologies and the extensive amount of relevant information for risk assessment that can be obtained from the genome [47]. On the one hand, advances in sequencing technologies have reduced the cost of genome sequencing. Consequently, it makes WGS economically feasible for companies that produce biopesticides, thus removing financial barriers [61]. On the other hand, WGS permits the identification of AMR genes, meets other data requirements such as unequivocal identification of microbial strains, and detects other critical traits, such as virulence factors and the production of toxic metabolites [31].

The WGS process for AMR starts with extracting DNA from the microorganism used as a microbial pesticide. Next, the DNA is sequenced, which may involve library construction and amplification for some next generation sequencing (NGS) platforms. The raw sequences are then assembled, and genome quality is assessed using bioinformatics tools. Finally, the genome is annotated to identify AMR genes [47]. The NGS tools most used for microbial WGS are Illumina (NextSeq and MiSeq), Oxford Nanopore Technologies (ONT: MinION and GridION), and Pacific Biosciences. Illumina platforms, use short read through a method called sequencing by synthesis with cyclic reversible termination [61]. ONT platforms, employ single-molecule real-time sequencing without relying on a clonal population of amplified DNA fragments. PacBio’s SMRT sequencing involves specialized flow cells containing thousands of picolitre wells with transparent bottoms called zero-mode waveguides (ZMWs). PacBio’s method focuses on single molecules, enabling continuous observation and recording of nucleotide incorporation, which enhances the accuracy and depth of genomic data [61].

Once the genome is sequenced, it must be analysed with different bioinformatic algorithms. The process involves *de novo* assembly [62], or aligning and comparison of the sequence reads to a reference genome, and quality control (QC) checks to assess the quality and quantity of raw sequenced reads (coverage), the assembly’s quality and to detect contaminant DNA [63]. Currently, a universally accepted standard for the bioinformatic analysis does not exist [47]. To identify AMR genes, the European guidelines (i.e., SANTE/2020/12260, EFSA 2021) recommend searching at least two maintained and curated databases, such as Comprehensive Antibiotic Resistance Database (CARD) [64–66], ResFinder [67] and Antibiotic Resistance Gene-ANNOTation (ARG-ANNOT) [68] using the minimum available threshold for length coverage. Queries should have at least 80% identity and 70% length coverage. CARD is a curated database that integrates diverse molecular and sequence data, structured through the Antibiotic Resistance Ontology (ARO) as a unifying framework. It facilitates the identification of putative antibiotic resistance genes in unannotated genomes. It offers various tools, such as the Resistance Gene Identifier (RGI), for predicting resistance genes in genomic and metagenomic data. It provides very detailed data [64]; however, it requires more bioinformatic expertise for detailed analysis compared to ResFinder and ARG-ANNOT.

ResFinder, a web-based method, is the most user-friendly of the three databases, it uses BLAST to identify acquired AMR genes in microbial genomes and is practical for quick applications [67]. ARG-ANNOT focuses on the annotation and classification of resistance genes, providing tools for sequence alignment and annotation, making it practical and user-friendly for annotation purposes [68].

The analysis should be performed and documented by the EFSA statement on the requirements for WGS analysis of microorganisms. The analysis should be presented in a table focusing on complete genes coding for resistance to antimicrobials. The table should include: (1) Gene identification. (2) Function of the encoded protein (3) Percentage of identity (4) E-value. Furthermore, regions upstream and downstream of the gene should be analysed to assess their potential transferability [31].

The use of WGS analyses is a huge improvement in the risk assessment of microorganisms, because it can identify information on the presence of AMR genes and mobile genetic elements indicating transferability, which might not have been detected in a literature-based risk assessment missing information on strain level. However, some considerations need to be made. For example, a combination of WGS analysis and phenotypic testing is mandatory. Otherwise, false positive results may occur in the case of pseudo genes, but false negative results are also possible because only previously known AMR mechanisms can be detected via WGS based on reference genomes [47]. In conclusion, ongoing research needs to be conducted for the further detection of AMR mechanisms, and it points out the importance of curation and the permanent updating of existing databases for increased sensitivity to AMR prediction via WGS.

**Table 2:** Approval criteria for antimicrobial resistance (AMR) according to SANTE/2020/12,260

Criteria			Approval decision
WGS (hit)	Presence of MGE	Phenotypic screening	
+	-	-	+
+	+	-	+
+	-	+	+
-	?	+	+*

+ positive; - negative;? unknown; * until more information is available

## Conclusion and outlook

Contrary to chemical active substances (AS), challenges in the field of regulatory approval procedure for microbial pesticides include the ability of microorganisms to multiply, their capacity to produce secondary metabolites, toxins, virulence factors, as well as their potential to transfer antimicrobial resistance (AMR). Notably, the transcription of genes associated with these traits may only occur under specific conditions, such as in the presence of a host. Additionally, two critical factors in the risk assessment of microorganisms are pathogenicity and infectivity, which must be thoroughly evaluated and excluded before microbial AS approval. Another significant challenge is the potential host specificity of microorganisms, which raises questions about the relevance of animal studies for assessing risks to human health.

The existing approach to human risk assessment is broadly protective but not always optimally tailored to the biological nature of microbial AS. This can lead to both under- and over-estimation of risk in different cases. Compared to chemical pesticides, which benefit from decades of methodological refinement and standardisation, microbial pesticide risk assessment is still developing. As the European Green Deal supports the use of biological and physical methods over conventional chemical methods, there is a clear need for further adaptation of assessment tools and frameworks to reflect the specificities of microbial agents more accurately and effectively.

NAMs can pave the way to overcome challenges and improve the risk assessment of microbial pesticides. Efforts have been made in this context, and whole genome sequencing (WGS) analysis together with in silico predictions, has already been applied as a NAM-based strategy in the risk assessment of microbial pesticides. This strategy is firstly used for taxonomic classification and strain identification, which serve as a base for a literature review. The literature review can retrieve information on pathogenicity and infectivity enabling the exclusion of microorganisms exhibiting these effects when sufficient data is available within a weight of evidence (WoE) approach. In such cases, this potentially allows for the waiver of the required animal studies. WGS analysis also aids in addressing additional effects of microorganisms required by regulations for microbial pesticides. It enables the identification of genes encoding for secondary metabolites, toxins, other virulence factors, and AMRs together with possible mobile genetic elements (MGE) responsible for transferability.

The application of NAMs is still at a very early stage with regard to human health risk assessment for microbial AS, but it has the potential to reduce the burden of certain data requirements. Notably, current approaches for acute toxicity testing to assess mammalian pathogenicity and infectivity (oral, dermal, and inhalation studies) remain highly animal-intensive, despite often yielding limited or negative findings for many microbial agents. These studies can also lack predictive value due to species-specific interactions and exposure routes that do not reflect human use scenarios. Repeated-dose and immunotoxicity testing also represent areas where traditional methods are resource-heavy but may add minimal value, particularly for microbial strains with a history of safe use. Prioritising the development and regulatory acceptance of NAMs in these specific areas could meaningfully reduce animal use and enhance the risk assessment process for microbial pesticides.

Another limiting factor to consider when examining the potential of NAMs in microbial AS risk assessment is the lack of “whole organism” perspective. Chemical AS assessments primarily rely on exposure data, toxicokinetics and metabolism assays to address this. These assays are of limited value for microbial risk assessment, however, due to the complex nature of the microbial AS. Therefore, exposure assessment focuses on the viability of the microorganism under various environmental conditions, including its persistence in soil, water, or on plants, and how it comes into contact with humans (e.g., oral, dermal or inhalation). The dose of viable organisms at the point of exposure is crucial, as microbial activity can vary over time. Toxicokinetics for microbial biopesticides involves understanding the potential for sensitisation or the production of secondary metabolites that could impact human health, both of which fall outside the scope of this review and are well reviewed by Paege et al. and Leme et al. (manuscript in preparation) [19, 20].

The IATA framework has gained popularity in the field of NGRA; however, reliability and interpretation criteria should be well-defined in order to draw appropriate conclusions about hazards and/or risks. For instance, in the regulatory field of microbial pesticides, utilizing WGS data requires access to well-curated databases to support robust in silico analyses. Furthermore, it is crucial to harmonize WGS data analysis and further develop prediction tools that are accessible, user-friendly, and highly reliable with high sensitivity. Once critical genes are identified, their functionality still needs to be verified through in vitro methods. For AMR genes, this can be done using classical growth inhibition zone assays; however, this is more challenging in the case of genes associated with secondary metabolites, toxins, and virulence factors. In this regard, and to reduce the use of animal testing, the advancement of non-animal methods, such as in vitro approaches based on cell culture systems and relevant biomarkers to predict the biological effects of microorganisms on human health, needs to be prioritized.

In essence, future research projects focusing on the development of NAMs are indispensable to the goal of performing risk assessment to human health free of in vivo studies.

## Data Availability

No datasets were generated or analysed during the current study.

## Abbreviations

AMR: Antimicrobial Resistance
AOP: Adverse Outcome Pathway
ARG-ANNOT: Antibiotic Resistance Gene-ANNOTation
ARO: Antibiotic Resistance Ontology
AS: Active Substance
CARD: Comprehensive Antibiotic Resistance Database
CLP: Classification, Labelling and Packaging
COVID-19: Coronavirus Disease 19
ECHA: European Chemicals Agency
EFSA: European Food Safety Authority
EU: European Union
FMDV: Foot and Mouth Disease Virus
GLP: Good Laboratory Practice
IATA: Integrated Approaches to Testing and Assessment
MDR: Multidrug-resistant
MGE: Mobile Genetic Elements
MIC: Minimum Inhibitory Concentration
MPCAs: Microbial Pest Control Agents
NAMs: New Approach Methodologies
NGRA: Next Generation Risk Assessment
NGS: Next Generation Sequencing
OECD: Organisation for Economic Co-operation and Development
ONT: Oxford Nanopore Technologies
PPP: Plant Protection Product
QC: Quality Control
QPS: Qualified presumption of safety
RGI: Resistance Gene Identifier
RNAi: RNA interference
TGAI: Technical Grade Active Ingredient
WGS: Whole Genome Sequencing
WoE: Weight of Evidence
ZNWs: Zero-mode waveguides
